# Novel dry pericardiocentesis: Transvenous puncture of the right ventricle with the back end of a 0.014-inch PTCA guidewire and a 1.8 Fr microcatheter

**DOI:** 10.3389/fcvm.2022.974601

**Published:** 2022-09-06

**Authors:** Hua-Di Qin, Hui Gao, Jie Gao, Lin Hou, Xiang-Seng Shao, Jing-Wei Tang, Chun-Chang Qin

**Affiliations:** ^1^Department of Anesthesia, The First Affiliated Hospital of Chongqing Medical University, Chongqing, China; ^2^Department of Ultrasound, The Affiliated Children's Hospital of Chongqing Medical University, Chongqing, China; ^3^Department of Cardiology, The First Affiliated Hospital of Chongqing Medical University, Chongqing, China; ^4^Department of Ultrasound, The People's Hospital of Shapingba District, Chongqing, China

**Keywords:** transvenous, dry pericardiocentesis, PTCA guidewire, microcatheter, right ventricle

## Abstract

**Background:**

Dry transthoracic pericardiocentesis is challenging and carries the risk of right ventricle (RV) or coronary artery injury. The RV can usually control bleeding automatically. For example, most perforations of the RV caused by pacemaker leads are treated without open surgery. Thus, we performed a transvenous puncture of the RV for dry pericardiocentesis with the back end of a 0.014-inch percutaneous transluminal coronary angioplasty (PTCA) guidewire and a 1.8 Fr microcatheter.

**Methods:**

The back end of a 0.014-inch PTCA guidewire within a 1.8 Fr microcatheter was used to transvenously punctured through the middle of the acute margin of the RV into the pericardial space in 12 Yorkshire swine and 5 beagles. PTCA balloons of different diameters were used to dilate the puncture holes for 15 min under anticoagulation in all the animals to assess the ability of the RV to control the bleeding. Then, for 3 days, the puncture hole was dilated by a 6 Fr catheter in 9 swine and 5 dogs.

**Results:**

The puncture was successful in all the animals. After withdrawal of the 2.5-mm balloon or the 6 Fr catheter, none of the animals exhibited pericardial effusion, as observed by echocardiography. There was no sustained ventricular arrhythmia or other complications. All the animals survived.

**Conclusion:**

Transvenous puncture of the right ventricle with the back end of a 0.014-inch PTCA guidewire and 1.8 Fr microcatheter may be feasible and have a good safety margin.

## Introduction

Dry pericardiocentesis may be associated with severe complications, including puncture or laceration of the cardiac and coronary arteries (1). Sosa introduced a wire-guided technique for dry pericardiocentesis (2). This technique is also complicated and is associated with some serious complications (3). The risks can be decreased by injecting fluid into the pericardial space to separate the visceral and parietal pericardium. The right atrial appendage and coronary sinus were used to inject fluid into the pericardial space for transvenous pericardiocentesis (1, 4), which reduced the risk of transthoracic dry pericardiocentesis. However, the atrial appendage is thin and poorly contractile and has been shown to be associated with a risk of hemorrhage. Transvenous puncture of the coronary sinus is a complicated and difficult procedure.

**Figure 1:**
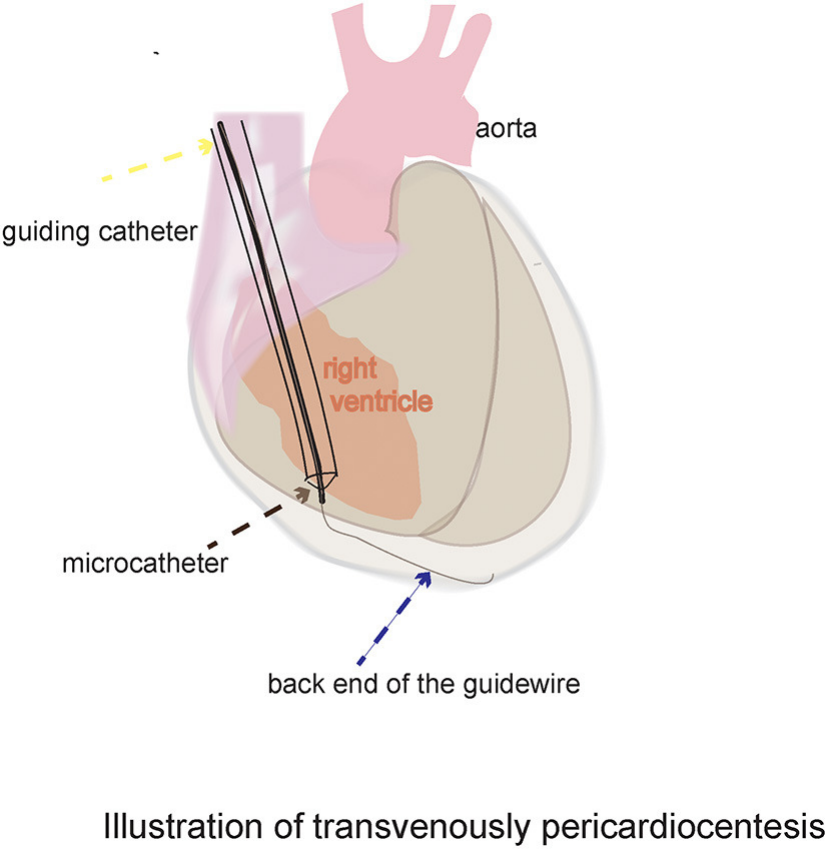
Schematic diagram of the novel dry pericardiocentesis procedure: With the support of the guiding catheter and microcatheter, the back end of the 0.014-inch guidewire was used to puncture through the middle of the acute margin of the right ventricle into the pericardial space.

Clinical experience suggests that the right ventricle (RV) has some capacity to control bleeding. For example, perforations of the RV caused by pacemaker leads are usually treated without open surgery (5–7), which suggests that the RV can spontaneously control bleeding after the withdrawal of leads with diameters of 2.3–3.0 mm. This ability may be related to the thick and contractile myocardium. These results support the idea that the RV may allow transvenous access to the pericardial space. However, according to textbook of pericardial interventions, transvenous puncture of the RV is not possible in humans because of the risk of severe hemorrhage (8). Indeed, severe hemorrhage is usually due to lacerations that occur when the heart beats against a sharp needle tip rather than from a needle puncture hole (3). If a flexible and blunt tool can be used to puncture the RV, it can move with the heartbeat, and the risk of laceration is decreased. The back end of a 0.018 inch guidewire, which is flexible and blunt, can be used to puncture through the RAA into the pericardial space. Because the wall of the RV is thicker than that of the RAA, whether the back end of a 0.014-inch guidewire can puncture through the wall of the RV remains unknown. The back end of the PTCA wire we used was much rougher than the front end of all the other wire. Thus, a transvenous puncture of the RV with the back end of a 0.014-inch PTCA guidewire and 1.8 Fr microcatheter was tested in swine and dogs (Figure 1). We also dilated the puncture hole with a PTCA balloon or 6 Fr catheter to evaluate the safety margin.

## Methods

The experimental protocol was approved by the Institutional Animal Care and Use Committee of the First Affiliated Hospital of Chongqing Medical University. All animals received humane care in accordance with the 1996 Guide for the Care and Use of Laboratory Animals recommended by the U.S. National Institutes of Health. This study used 12 Yorkshire swine (mean weight, 37.4 ± 6.7 kg; range: 30–54 kg) and five beagles (weight range: 14–15 kg). For preanesthesia, each animal was given 0.04 mg/kg atropine i.m.; swine received 0.6 mg/kg midazolam i.m., and dogs received 1.2 mg/kg midazolam i.m. Anesthesia was maintained with intravenous propofol (60–300 mg/h). The 6 Fr sheath was implanted into the jugular vein using the Seldinger technique under ultrasound guidance. Arterial blood pressure was monitored through a femoral artery sheath. The electrocardiograms were monitored. Unfractionated heparin (80 IU/kg bolus, followed by a 20 IU/kg/h bolus during the intervention) was administered to all the animals to increase the risk of bleeding. The mean activated clotting time was 213.0 ± 36.7 s before the transventricular balloon was withdrawn into the right ventricle. The intervention was performed under fluoroscopic guidance. The middle of the acute margin of the RV was selected as the puncture site. A 6 Fr SAL 0.75 guiding catheter (Medtronic Inc., Minneapolis, USA) was navigated toward the RV. The catheter tip was directed toward the right border of the heart shadow between the ventricular apex and annulus, with a left anterior oblique projection (LAO) of 30°, with the animals in the left recumbent position. Then, the back end of a 0.014-inch Sion guidewire (Asahi Intec, Seto, Japan) within a 1.8 Fr Finecross microcatheter (Terumo Corporation, Tokyo, Japan) was advanced through the guiding catheter (Supplementary Figure 1). The back end of the guidewire was used to puncture through the RV into the pericardial space (Supplementary Video 1). Then, the microcatheter was advanced over the guidewire into the pericardial space (Supplementary Video 2). To verify that the tip of the microcatheter was in the pericardial space, approximately 2 ml of Ultravist-370 contrast (Bayer Pharma, Berlin, Germany) was injected *via* the microcatheter (Supplementary Video 3).

The puncture hole was dilated with a PTCA balloon in all 12 swine and five dogs to investigate the capacity of the RV wall to control bleeding. Balloons of 2.0 × 15 mm, 2.5 × 23 mm, and 3.0 × 23 mm (Abbott Vascular, Santa Clara, USA) were successively applied to dilate the puncture hole in the RV. The balloon was inflated to a nominal pressure for 15 min. Contrast was injected through the guiding catheter to confirm the position of the balloon in the ventricular wall (Supplementary Video 4). Fifteen minutes after the balloon was withdrawn, echocardiography was performed to check for pericardial effusion.

A 6 Fr guiding catheter was used to dilate the RV puncture hole for 3 days in nine swine and five dogs for evaluation of the ability to control bleeding after long-term dilation. The guiding catheter was selected because it is easier to be fixed, and the diameter of the entire catheter body is the same. Therefore, the unintentional movement of the catheter would not affect the dilation diameter. First, a 2.8 Fr Cosair microcatheter (Asahi Intecc, Seto, Japan) was inserted into a 5 Fr MP (Cordis Corporation, Warren, USA) diagnostic catheter (Supplementary Figure 2), and the assembly was advanced over the guidewire into the pericardial space. Then, a 0.035-inch Radifocus wire (Terumo Corporation) was navigated into the pericardial space *via* the MP catheter. Subsequently, a 120-cm-long 5 Fr MP diagnostic catheter was inserted into a 6 Fr JR 3.5 (Medtronic Inc.) guiding catheter (Supplementary Figure 3). The assembly was carefully advanced along the 0.035-inch wire into the pericardial space. The MP catheter was withdrawn, and the 0.035-inch wire was left to prevent injury caused by the catheter tip. Finally, the wire and JR 3.5 guiding catheter were left in place to dilate the puncture hole for 3 days. Cefoxitin sodium (0.1 g/kg, i.m.) was administered once a day. Three days later, the catheter and wire were withdrawn, and echocardiography was performed to monitor pericardial effusion. Two days after the procedure, the animals were killed, and their hearts were inspected.

Data were analyzed using SPSS (v21.0, IBM) and are reported as the mean ± standard deviation unless otherwise indicated.

## Results

**Figure 2:**
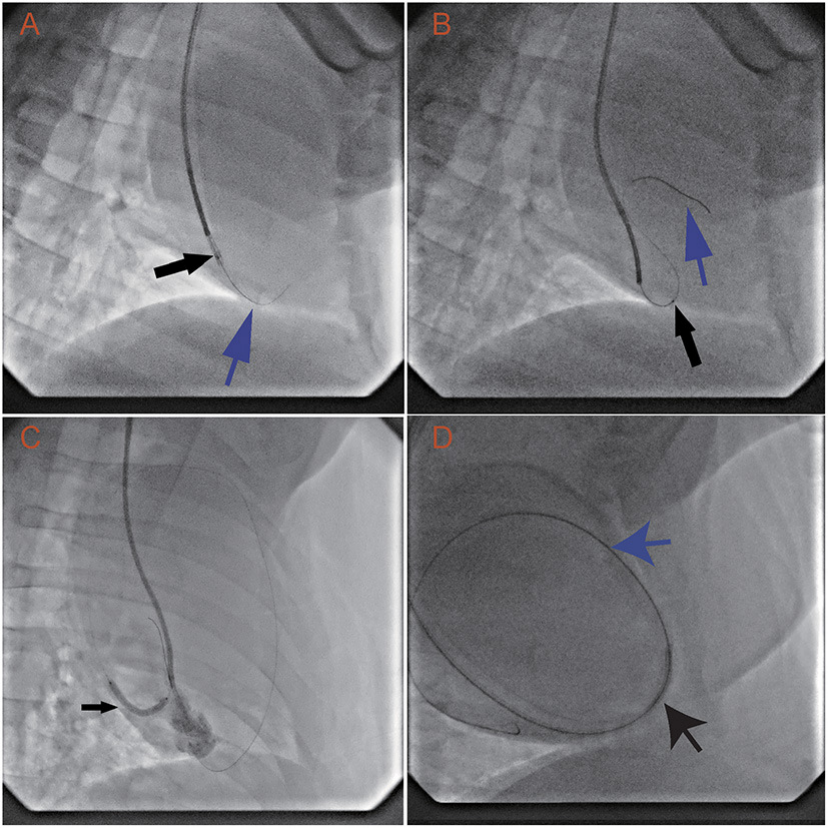
Transvenous puncture of the acute margin of the RV and confirmation of its safety margin by dilation of the puncture hole. The procedure uses an LAO projection of 30°, while the animals are in a left recumbent position. **(A)** With the support of the 1.8 Fr Finecross microcatheter (black arrow) and 6 Fr SAL 0.75 guiding catheter, the back end of the Sion wire (blue arrow) is used to puncture the middle of the acute margin of the right ventricle. **(B)** After the 1.8 Fr Finecross microcatheter (black arrow) is advanced into the pericardial space, the front end of the wire (blue arrow) is navigated into the pericardial space. **(C)** The puncture hole is dilated with a 2.5 × 23 mm balloon (12 atm × 15 min). **(D)** A 6 Fr JR 3.5 guiding catheter, with a 0.035-inch wire, is placed through the right ventricular wall into the pericardial space.

In all animals, the RV was successfully punctured with the back end of a 0.014-inch guidewire and 1.8 Fr microcatheter. The guidewire and microcatheter easily punctured through the RV into the pericardial space. Once the back end of the guidewire contacted the ventricular wall, puncture into the pericardial space took 7.8 ± 3.9 s. The guidewire tracked along but was not used to puncture the parietal pericardium (Figure 2A). The microcatheter was advanced along the guidewire into the pericardial space in several seconds (Figure 2B).

The RV can spontaneously control bleeding after dilation with a 2.5-mm balloon for 15 min under anticoagulation. The first two swine underwent serial dilation with 2-, 2.5-, and 3-mm balloons and showed no observable signs of pericardial effusion. However, the third swine displayed minor pericardial effusion (echocardiography showed effusion with a depth of 4 mm in diastole) after dilation with a 3.0-mm balloon. The animal's circulation was not compromised, and drainage was not needed. To provide a solid safety margin, the 3-mm balloon was not tested in the remaining animals. Then, the remaining swine and all the dogs received dilation with a 2.5-mm balloon only (Figure 2C). After the 2.5-mm balloon was withdrawn from all the animals, echocardiography was performed and showed no signs of pericardial effusion. These results suggest that the acceptable dilation size is no more than 2.5 mm and that the RV wall can spontaneously control bleeding under anticoagulation.

The RV also has the capacity to control bleeding after a long dilation time. The 6 Fr catheter was placed in the pericardial space *via* the RV wall puncture hole (Figure 2D) in nine swine and five dogs. Three days later, the catheter was withdrawn. Monitoring *via* echocardiography did not show any signs of pericardial effusion. The hearts were inspected after the procedure. The pericardial spaces were clean, and there were small (~1 mm), dull, red plugs in the puncture holes.

No complications were found to be associated with pericardial access. All the animals survived the experiments. On occasion, ventricular premature beats and nonsustained ventricular tachycardia were observed. These events were resolved after manipulation of the guiding catheter or wire was discontinued.

## Discussion

The back end of a 0.014-inch PTCA guidewire and 1.8 Fr microcatheter were used to easily puncture through the RV wall into the pericardial space. The RV automatically controlled bleeding. After dilation for 15 min with a 2.5-mm diameter balloon or dilation for 3 days with a 6 Fr catheter, there was no observable effusion on echocardiography after device withdrawal. This method has a good margin of safety.

RV access has been adopted for ventricular septal defect (VSD) occlusion in humans. Global experience shows that the procedure is feasible, safe and effective (9). The puncture hole needs to be sutured during VSD occlusion procedures that occur under direct vision. However, the puncture hole cannot be sutured in transvenous pericardiocentesis, and thus, spontaneous bleeding control is needed.

The RV wall spontaneously controls bleeding after dilation with a 2.5-mm balloon under anticoagulation, which is consistent with the fact that most perforations of the right ventricle are caused by pacemaker leads and can be treated by simply transvenous withdrawal and/or pericardiocentesis, without open surgery sutures. The diameters of the leads are usually 2.3–3 mm (6, 10), so some leads are larger than the 2.5-mm balloon. The difference may result from anticoagulation. This scenario is also consistent with the clinical experience of dry pericardiocentesis. RV myocardial punctures are frequent, but most do not require open surgery (11). This indicates that myocardial puncture holes recoil and control bleeding. The RV myocardium is thick and contractile, with a complex three-dimensional network of myofibers in multiple helical arrangements. On the epicardial and subendocardial surfaces, the myofibers are circumferentially and longitudinally oriented, respectively (12). They can compress the hole from different directions, which is helpful for controlling bleeding.

The middle of the acute margin of the RV was selected as the puncture site. This site is easy to reach with a catheter *via* jugular vein access, and there are no large vessels nearby. Most importantly, different areas of the RV wall have variable anatomic characteristics. Compared with the apex and outflow tract near the pulmonary valve, which are 1–2 mm, the middle of the acute margin of the RV wall is 3–5 mm (13). In addition, the epicardial myofibers in this area are perpendicular to the subendocardium layer. This is helpful for closing the puncture hole during myocardial contraction. In contrast, the epicardial myofibers spiral into the endocardium of the apical area (12). Collectively, these results suggest that the middle of the acute margin wall has a better capacity for bleeding control.

Although the back end of a guidewire is flexible and blunt, it can easily puncture the RV wall. The penetration ability of the guidewire can be dramatically increased with the support of a microcatheter and guiding catheter (14). This technique is widely used in percutaneous coronary interventions for chronic total occlusion. After puncturing the ventricular wall, the penetration ability of the guidewire weakens because it has less support from the microcatheter and guiding catheter, and it will not puncture the parietal pericardium. The guidewire dimensions and flexibility allow it to naturally curve over the surface of the heart.

Compared with a needle, the back end of a guidewire is safe for pericardiocentesis. It is blunt and flexible, and it can follow the movement of a beating heart when it crosses the RV to minimize the risk of laceration. Needles are associated with a risk of myocardial tearing. Although entry of an 18-gauge (1.3-mm outer diameter) needle into the RV is considered benign (15), tamponade occasionally occurs in actual practice. It is important to recognize that serious bleeding complications are due to lacerations that occur from the heart beating against a sharp needle tip rather than from the direct needle puncture hole (3). Torsional cardiac and translational respiratory motion exacerbates the risk of laceration as the heart beats against a sharp needle tip. For example, acupuncture needles with diameters of 0.3–0.5 mm may accidentally cause lacerations of several mm in the RV wall (16–18). Furthermore, the guidewire is advanced through the guiding catheter, which makes it easier to manipulate than a needle. For example, the long and rigid Brockenbrough needle, which is frequently used for atrial septal puncture, has a long learning curve, and the rate of complications associated with it ranges from 0.0 to 6.7% (19).

This method may be useful for specific clinical conditions, such as diagnostic sampling in patients with minor pericardial effusion and local cardiac drug delivery in the absence of pericardial effusion. For patient safety, the RV can be used as an access for a 1.8 Fr microcatheter but cannot serve as an access for a sheath or ablation catheter, which may cause damage. In the field of electrophysiology, this method can be used to convert dry pericardiocentesis to wet pericardiocentesis by injecting fluid into the pericardial space. Then, transthoracic pericardiocentesis can be safely performed. It may be applied for epicardial ablation, ligation of the left atrial appendage, and pacemaker lead implantation. In the pre-experiment, transthoracic pericardiocentesis was easily performed after injecting 80 ml of diluted contrast into the dry pericardium (Supplementary Figure 4, Supplementary Video 6). Furthermore, previous studies reported that transthoracic pericardiocentesis could be performed easily after injecting fluid or CO2 into the pericardium (1, 3, 20), so we did not perform it in this experiment.

There are several major limitations of this study. First, the procedure was tested in only a small number of animals. Second, there are differences between human and animal hearts. The safety and feasibility of this approach need to be demonstrated in human subjects. Third, the observation time was a short period of only several days. Fourth, the use of the back end of the PTCA wire is off label, so it needs to be evaluated and approved by the FDA in the future.

In summary, transvenous puncture of the right ventricle with the back end of a 0.014-inch PTCA guidewire and 1.8 Fr microcatheter may be a feasible and safe method for dry pericardiocentesis. It may be used to convert dry pericardiocentesis to wet transthoracic pericardiocentesis.

## Data availability statement

The original contributions presented in the study are included in the article/Supplementary material, further inquiries can be directed to the corresponding author/s.

## Ethics statement

The animal study was reviewed and approved by the Institutional Animal Care and Use Committee of the First Affiliated Hospital of Chongqing Medical University. All animals received humane care in accordance with the 1996 Guide for the Care and Use of Laboratory Animals recommended by the U.S. National Institutes of Health.

## Author contributions

HG, H-DQ, and JG performed the animal experiments. X-SS, LH, and J-WT gathered and contributed to the interpretation of the data. C-CQ designed the experiment and wrote the article and they acknowledge that they have exercised due care in ensuring the integrity of the work. All authors have read and approved the manuscript for submission and have made substantial contributions.

## Funding

This work was supported by the National Natural Science Foundation of China (No. 82070523 to C-CQ), Chongqing Science and Technology Bureau (No. cstc2019jscx-msxmX0307 to C-CQ), and Chongqing Health Commission (No. 2020msxm113 to C-CQ).

## Conflict of interest

The authors declare that the research was conducted in the absence of any commercial or financial relationships that could be construed as a potential conflict of interest.

## Publisher's note

All claims expressed in this article are solely those of the authors and do not necessarily represent those of their affiliated organizations, or those of the publisher, the editors and the reviewers. Any product that may be evaluated in this article, or claim that may be made by its manufacturer, is not guaranteed or endorsed by the publisher.

## Supplementary material

Supplementary Figure 1 **(A)** Within the Finecross microcatheter, the Sion wire is advanced through the 6 Fr SAL 0.75 guiding catheter. **(B)** Enlarged image of the tip of the assembly. Black arrow: SAL 0.75 guiding catheter. Yellow arrow: Finecross microcatheter. Blue arrow: The back end of the Sion wire.

Supplementary Figure 2 **(A)** The Sion wire within the Cosair microcatheter is advanced along the 5 Fr MP catheter. **(B)** Enlarged image of the tip of the assembly. Black arrow: MP catheter. Yellow arrow: Cosair microcatheter. Blue arrow: The front end of the Sion wire.

Supplementary Figure 3 **(A)** A 5 Fr MP catheter within the 6 Fr JR 3.5 guiding catheter is advanced along the 0.035” wire. **(B)** Enlarged image of the tip of the assembly. Blue arrow: MP catheter. Yellow arrow: Cosair microcatheter. Blue arrow: The front end of the 0.035-inch wire.

Supplementary Figure 4 A wet transthoracic pericardiocentesis procedure: Transthoracic pericardiocentesis was performed after diluted contrast agent was injected *via* the microcatheter, which was going through the right ventricle into the pericardial space. Yellow arrow: the injected diluted contrast in pericardium space, White arrow: the tip of the microcatheter in the pericardium space, blue arrow: the wire in the pericardium space, black arrow: the needle is puncturing through thorax.

Supplementary Video 1 With the support of the 1.8 Fr Finecross microcatheter (its marker is the black point at the tip of the 6Fr SAL 0.75 guiding catheter) and guiding catheter, the back end of the 0.014-inch Sion wire is used to puncture the middle of the acute margin of the right ventricle.

Supplementary Video 2 The 1.8 Fr Finecross microcatheter (the black point moving along the wire) is advanced along the wire into the pericardial space.

Supplementary Video 3 The contrast is injected through the microcatheter (the black point) to confirm the position of the microcatheter.

Supplementary Video 4 The puncture site is dilated with a 2.5 × 23 mm balloon at 12 atm, and some contrast is injected into the right ventricle to confirm the position of the balloon.

Supplementary Video 5 A 6 Fr guiding catheter with a 0.035-inch wire is placed through the ventricular wall into the pericardial space.

Supplementary Video 6 A wet transthoracic pericardiocentesis procedure was performed after the diluted contrast agent was injected *via* the microcatheter, which was navigated through the right ventricle into the pericardial space.

## Abbreviations

PTCA: percutaneous transluminal coronary angioplasty
RV: right ventricle
RAA: right atrial appendage
LAO: left anterior oblique projection
VSD: ventricular septal defect.
