# Supplementary figures and images for: Novel dry pericardiocentesis: Transvenous puncture of the right ventricle with the back end of a 0.014-inch PTCA guidewire and a 1.8 Fr microcatheter
Source: Front Cardiovasc Med. 2022 Sep 6;9:974601. doi: 10.3389/fcvm.2022.974601 (PMC9485541; doi:10.3389/fcvm.2022.974601)

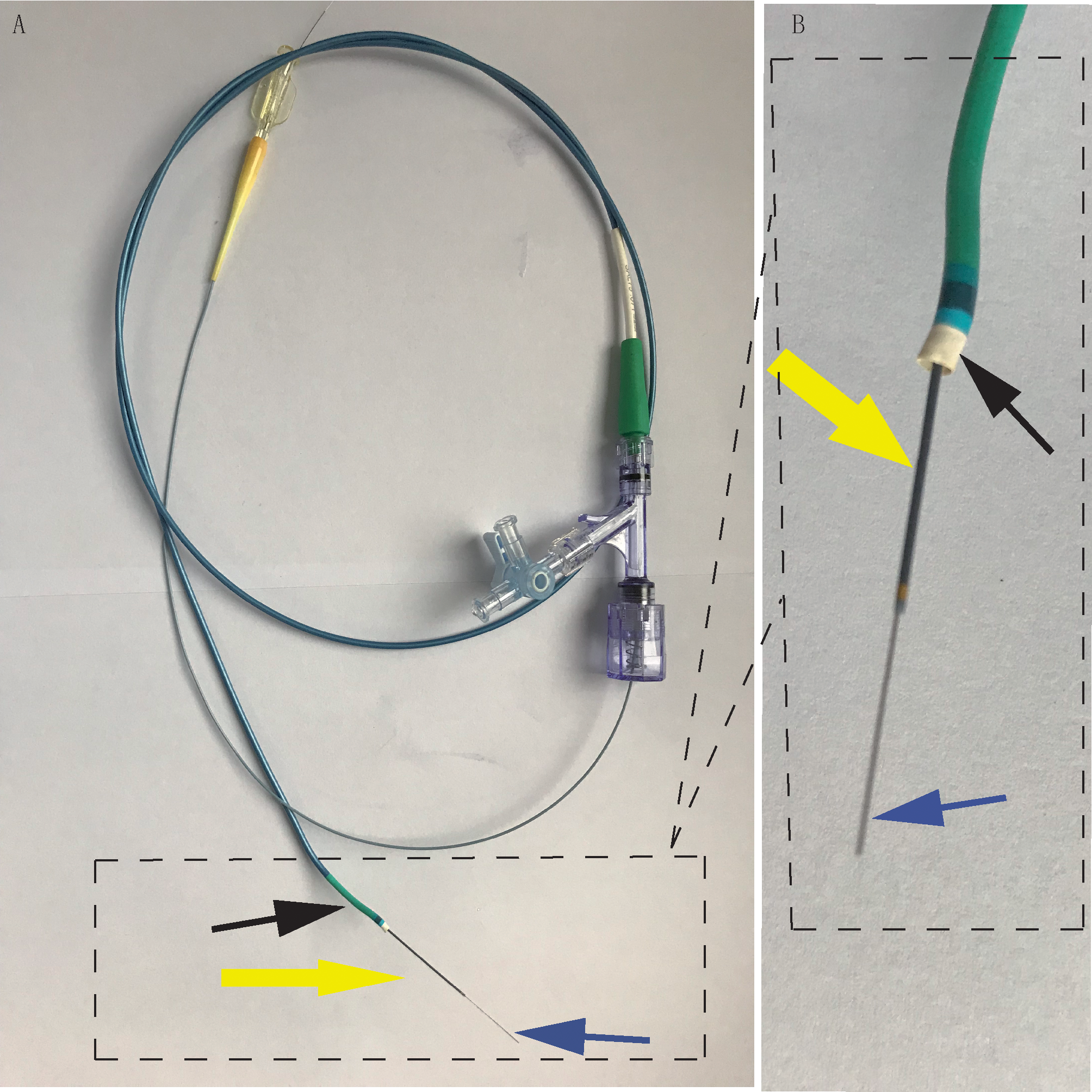

Supplement: Supplementary Figure 1 — (A) Within the Finecross microcatheter, the Sion wire is advanced through the 6 Fr SAL 0.75 guiding catheter. (B) Enlarged image of the tip of the assembly. Black arrow: SAL 0.75 guiding catheter. Yellow arrow: Finecross microcatheter. Blue arrow: The back end of the Sion wire. [file Image_1.TIF]

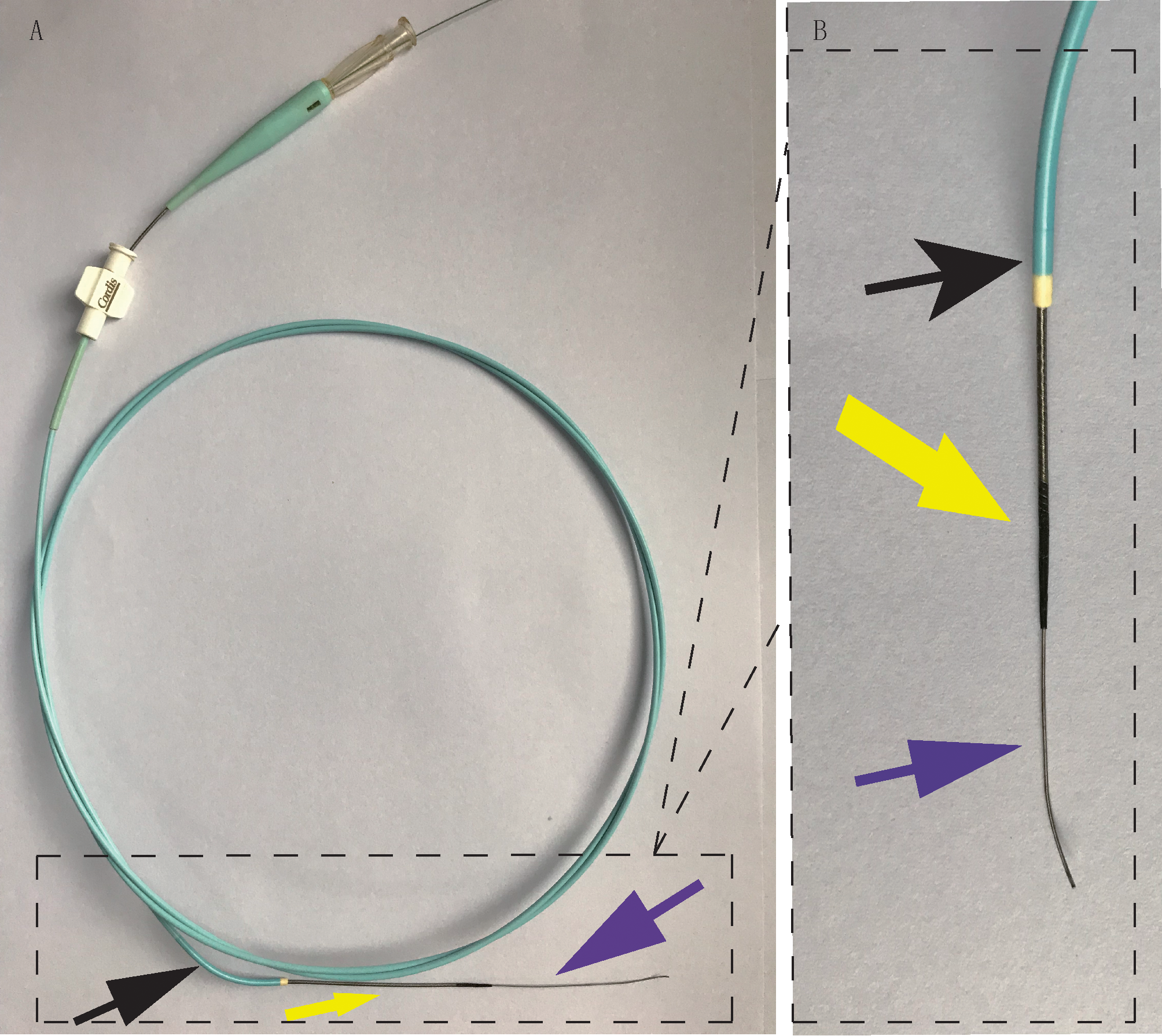

Supplement: Supplementary Figure 2 — (A) The Sion wire within the Cosair microcatheter is advanced along the 5 Fr MP catheter. (B) Enlarged image of the tip of the assembly. Black arrow: MP catheter. Yellow arrow: Cosair microcatheter. Blue arrow: The front end of the Sion wire. [file Image_2.TIF]

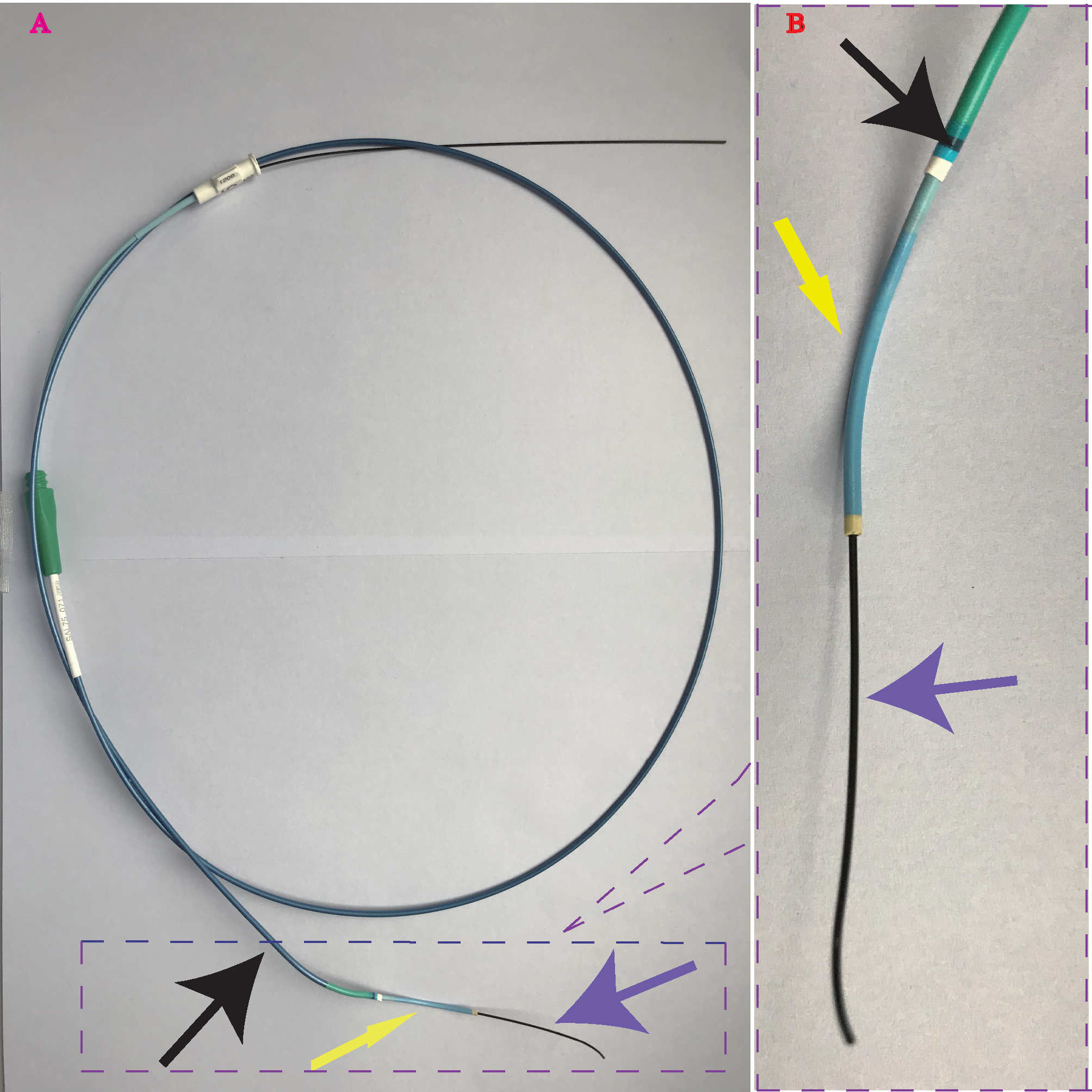

Supplement: Supplementary Figure 3 — (A) A 5 Fr MP catheter within the 6 Fr JR 3.5 guiding catheter is advanced along the 0.035” wire. (B) Enlarged image of the tip of the assembly. Blue arrow: MP catheter. Yellow arrow: Cosair microcatheter. Blue arrow: The front end of the 0.035-inch wire. [file Image_3.TIF]

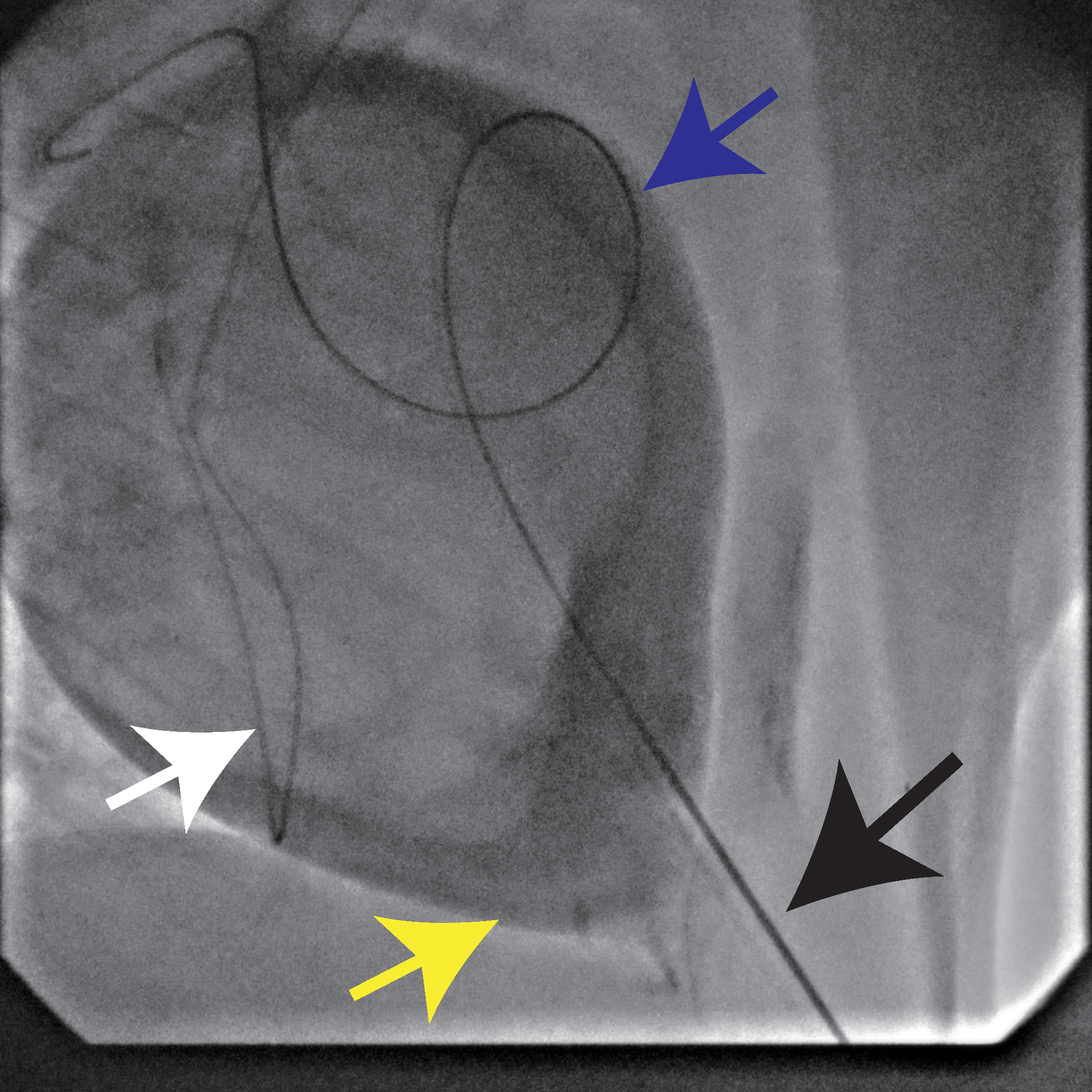

Supplement: Supplementary Figure 4 — A wet transthoracic pericardiocentesis procedure: Transthoracic pericardiocentesis was performed after diluted contrast agent was injected via the microcatheter, which was going through the right ventricle into the pericardial space. Yellow arrow: the injected diluted contrast in pericardium space, White arrow: the tip of the microcatheter in the pericardium space, blue arrow: the wire in the pericardium space, black arrow: the needle is puncturing through thorax. [file Image_4.TIF]
